# Anti-cancer activity of Chaga mushroom (*Inonotus obliquus*) against dog bladder cancer organoids

**DOI:** 10.3389/fphar.2023.1159516

**Published:** 2023-04-19

**Authors:** Amira Abugomaa, Mohamed Elbadawy, Yusuke Ishihara, Haru Yamamoto, Masahiro Kaneda, Hideyuki Yamawaki, Yuta Shinohara, Tatsuya Usui, Kazuaki Sasaki

**Affiliations:** ^1^ Laboratory of Veterinary Pharmacology, Department of Veterinary Medicine, Faculty of Agriculture, Tokyo University of Agriculture and Technology, Tokyo, Japan; ^2^ Faculty of Veterinary Medicine, Mansoura University, Mansoura, Egypt; ^3^ Department of Pharmacology, Faculty of Veterinary Medicine, Benha University, Moshtohor, Egypt; ^4^ Laboratory of Veterinary Anatomy, Department of Veterinary Medicine, Faculty of Agriculture, Tokyo University of Agriculture and Technology, Tokyo, Japan; ^5^ Laboratory of Veterinary Pharmacology, School of Veterinary Medicine, Kitasato University, Aomori, Japan; ^6^ Pet Health & Food Division, Iskara Industry Co., Ltd., Tokyo, Japan

**Keywords:** Chaga mushrooms, *Inonotus obliquus*, bladder cancer, organoids, CSCs, cell cycle, apoptosis, c-Myc

## Abstract

Despite its disadvantages, chemotherapy is still commonly used for the treatment of bladder cancer (BC). Developing natural supplements that can target cancer stem cells (CSCs) which cause drug resistance and distant metastasis is necessary. Chaga mushrooms are popular to have several health-promoting and anti-cancer potentials. Organoid culture can recapitulate tumor heterogeneity, epithelial environment, and genetic and molecular imprints of the original tissues. In the previous study, we generated dog bladder cancer organoids (DBCO) as a novel experimental model of muscle-invasive BCO. Therefore, the present study aimed to examine the anti-tumor potentials of Chaga mushroom extract (Chaga) against DBCO. Four strains of DBCO were used in the present study. Treatment with Chaga inhibited the cell viability of DBCO in a concentration-dependent way. Treatment of DBCO with Chaga has significantly arrested its cell cycle and induced apoptosis. Expression of bladder CSC markers, *CD44*, *C-MYC*, *SOX2*, and YAP1, declined in the Chaga-treated DBCO. Also, Chaga inhibited the phosphorylation of ERK in DBCO. Expression of downstream signals of ERK, *C-MYC,* and *Cyclins* (*Cyclin-A2*, *Cyclin-D1*, *Cyclin-E1*, and *CDK4*) was also inhibited by Chaga in DBCO. Interestingly, the combinational treatment of DBCO with Chaga and anti-cancer drugs, vinblastine, mitoxantrone, or carboplatin, showed a potentiating activity. *In vivo*, Chaga administration decreased tumor growth and weight of DBCO-derived xenograft in mice with the induction of necrotic lesions. In conclusion, Chaga diminished the cell viability of DBCO by inhibiting proliferation-related signals and stemness conditions as well as by arresting the cell cycle. Collectively, these data suggest the value of Chaga as a promising natural supplement that could potentiate the effect of adjuvant chemotherapy, lower its adverse effects, and thus, limit the recurrence and metastasis of BC.

## 1 Introduction

Carcinoma of the bladder (BC) is a global health issue with more than 200000 new fatalities as indicated by Global Cancer Statistics 2020 (Siegel et al., 2020; Sung et al., 2021). In humans, the disease occurs in two forms. The first, luminal papillary BC, is more common, superficial, low-grade, and less metastatic with a good prognosis. The second, muscle-invasive BC (MIBC), is less common (approximately 25%–30% of the cases), more complex, and with high stemness, metastasis, therapy resistance, and epithelial-mesenchymal transition (EMT), and bad prognosis (Rentsch et al., 2017) (Elbadawy et al., 2022). However, in dogs, the second form of BC (MIBC) is more common (≥90% of the cases) (Sommer et al., 2018), highly resistant to therapy (Mutsaers et al., 2003) (Fulkerson and Knapp, 2015), and with a bad prognosis because of its common incidence in the bladder trigone hindering its complete surgical resection (Sommer et al., 2018). Several studies showed that the BC of dogs largely resembles the BC of humans in terms of etiology, pathogenesis, symptoms, histopathology, genetic profile, and treatments (Knapp et al., 2014; Dhawan et al., 2015; Fulkerson and Knapp, 2015; Shapiro et al., 2015; Maeda et al., 2018; Elbadawy et al., 2019). Thus, the BC dog was considered a valuable research model for human MIBC (Sommer et al., 2018; Elbadawy et al., 2019; Abugomaa et al., 2022).

To date, the first-line treatment for MIBC is radical cystectomy (transurethral resection) followed by adjuvant chemoradiotherapy administration (Knapp et al., 2014; Fulkerson and Knapp, 2015). However, the side effects as well as the development of resistance to chemotherapy are the main problems leading to failure of treatment and recurrence of BC. Therefore, introducing new supporting herbal therapy with anticancer properties may provide synergism and decrease the chemotherapy-associated side effects and thus will be very beneficial to cancer patients.

Chaga mushrooms (*Inonotus obliquus*) are a whitish-rot fungus that belongs to the *Hymenochaetaceae* family and grows on birch tree roots in several countries (Lee et al., 2008). Chaga mushrooms are popular to have several health-promoting potentials such as anti-microbial, anti-inflammatory, antioxidant, and antitumor effects (Balandaykin and Zmitrovich, 2015). Therefore, Chaga mushrooms extract (Chaga) is immensely used as a traditional medicament in Asia and Eastern Europe (Balandaykin and Zmitrovich, 2015). Recently, several reports revealed that Chaga has cytotoxic effects against several types of cancer cells such as sarcoma (Chung et al., 2010), lung adenocarcinoma (Baek et al., 2018), colon cancer (Lee et al., 2009), melanoma (Youn et al., 2009) as well as hepatocellular carcinoma (Youn et al., 2008). Therefore, Chaga is a widely consumed botanical supplement by cancer patients (Buckner et al., 2018). Despite growing evidence of antitumor potentials exhibited by the Chaga and its components (Shashkina et al., 2006; Song et al., 2013), the underlying mechanisms are still hazy and the impact of Chaga on cancer prevention is not well understood. This may be due to most of these studies have been conducted on cell lines (Szychowski et al., 2021).

The three-dimensional (3D) culture model (organoids) is an exceptional and recently-developed technology in which the patient-derived epithelial cells are isolated from tissues and grown in a gel scaffold and a culture medium containing growth factors that enhance stem cell properties (Elbadawy et al., 2019). Organoids can thus recapitulate the detailed structure, functions, and features of their original tissues such as self-renewal, organization, and differentiation as well as genetic and molecular imprints (Lancaster and Knoblich, 2014; Elbadawy et al., 2019; Elbadawy et al., 2021a). The recent advances in organoid model revealed substantial promise in biological and translational investigations to evolve recent personalized therapies, particularly for cancer (Lancaster and Knoblich, 2014; Weeber et al., 2017; Abugomaa and Elbadawy, 2020; Elbadawy et al., 2021b; Elfadadny et al., 2021; Yoshida et al., 2022). In a previous article, we generated a new experimental model for MIBC using a dog BC organoid culture (Elbadawy et al., 2019). This model recapitulated several features of MIBC in humans such as histopathological characteristics, drug sensitivity, and gene expression profiles (Elbadawy et al., 2019).

Chaga is used by many cancer patients as a complementary medical supplement during chemotherapy or radiotherapy. However, based on our knowledge, there are no published data regarding the effects or molecular targets of Chaga in BC patients. Therefore, in the current study, we examined the possible antineoplastic effects of Chaga using our previously established model of patient-derived DBCO and demonstrated the underlying molecular mechanism.

## 2 Materials and methods

### 2.1 Chaga mushroom

Chaga was obtained from the Iskra Industry Co., Ltd (Nihonbashi, Chuo-ku, Tokyo, Japan). Briefly, to get Chaga, raw *I. obliquus* was washed, dried at 60°C, and fermented. Then the material was extracted by water. The solid residue was filtered off and the filtrate was concentrated on a rotary evaporator which was then vacuum dried at 80°C to constant mass. After fine powdering, Chaga was yielded. From 1,000 g of raw *I. obliquus*, 100 g of *Chaga* was obtained (10% of raw material). Chaga was suspended in dimethyl sulfoxide (DMSO, Fujifilm Wako Pure Chemical Co., Tokyo, Japan) to prepare stocks of 10 mg/ml and kept at −80°C. The working concentration of Chaga was adjusted to 25, 50, 75, and 100 μg/ml using culture media.

### 2.2 Anti-cancer agents, antibodies, and kits

Anti-cancer agents utilized in the current study were vinblastine sulfate, mitoxantrone hydrochloride (Cayman Chemicals, Ann Arbor, MI, United States), and carboplatin (Fujifilm WAKO Pure Chemical Co.). The primary antibodies were obtained as follows: T-extracellular signal-regulated kinase (ERK); P-ERK (Cell Signaling Technology Inc., Danvers, Massachusetts, United States); and total-VCP (GeneTex, Inc., Irvine, CA, United States). The Horseradish peroxidase (HRP) conjugated goat anti-rabbit IgG (Millipore, Temecula, CA, United States) was used as a secondary antibody. The TUNEL staining system (DeadEnd Fluorometric Terminal deoxynucleotidyl transferase dUTP nick end labeling system) was obtained from Promega (Madison, MI, United States) and the PCR primers were from FASMAC (Kanagawa, Japan).

### 2.3 BC organoid culture

In the current investigation, DBCO was produced using aseptically obtained urine samples from four dogs with BC illness. The samples were transferred immediately in a cooled shipping medium to our laboratory. Four strains of DBCO were generated as mentioned earlier in our previous articles (Elbadawy et al., 2019; Elbadawy et al., 2021b; Elbadawy et al., 2022). The details of culture conditions, medium, supplements, growth factors, processing, and passages of the DBCO were the same. Concisely, the culture medium was Advanced DMEM including 50% Wnt, R-spondin, and Noggin conditioned medium; 100 μg/mL Primocin; 10 mM nicotinamide; 1% GlutaMax; and 1 mM N-Acetyl-L-cysteine (Thermo Fisher Scientific, Waltham, MA, United States); 500 nM A83-01 (Adooq Bioscience, Irvine, CA, United States); and 50 ng/mL mouse EGF (PeproTech, Rocky Hill, NJ, United States). All dog owners provided written informed consent for the current study, and all experimental procedures were carried out following the guidelines of the Institute Animal Care and Use Committee of Tokyo University of Agriculture and Technology (Approval number: 0020007). Table 1 includes sample information.

**TABLE 1:** Sample information.

Case ID	Age (year)	Breed	Sex	Sampling date	Muscle-invasiveness	Prior therapy
BC21031	11	Toy poodle	Castrated male	8/9/2021	Muscle-invasive	None
BC21043	13	Toy poodle	Castrated male	11/10/2021	Muscle-invasive	None
BC21014	11	Mix	Female	4/9/2021	Muscle-invasive	None
BC22056	12	Chihuahua	Male	10/4/2022	Muscle-invasive	None

### 2.4 Cell viability screening

The effect of Chaga on DBCO cell viability was carried out as previously stated (Elbadawy et al., 2019; Elbadawy et al., 2021b). Briefly, 2 × 10^3^ cells/well of each DBCO were cultured in 10 µl of Matrigel in a 96-well culture plate with culture media. Twenty-4 h later, the organoid cells were treated with Chaga at 25, 50, 75, and 100 μg/ml (Chung et al., 2010; Lemieszek et al., 2011) and incubated at 37°C for 72 h. Further, the potentiating effects of Chaga on the sensitivity of DBCO to commonly used anti-cancer drugs were also assessed, where the effects of vinblastine, mitoxantrone, or carboplatin at different concentrations (Elbadawy et al., 2019) were assessed alone or simultaneously combined with Chaga (25 μg/ml) on cell viability of two strains of DBCO. The viability of BC cells was evaluated by Prestoblue assay kit (Thermo Fisher Scientific) and the microplate reader (TECAN, Seestrasse, Switzerland) was used to quantify the data. Representative phase contrast images were taken by a light microscope (BX-52; Olympus, Tokyo, Japan), and data from the microplate reader were plotted using Sigma Plot software 14.5 (Systat Software Inc., San Jose, CA, United States).

### 2.5 Flow cytometry

Analysis of the cell cycle was performed to recognize cell clusters in various phases of the cell cycle following Chaga treatment. Briefly, DBCO cells (2 × 10^5^/well) were embedded in Matrigel and seeded in 24-well plates. Twenty-4 h later, the organoid cells were treated with DMSO or Chaga (100 μg/ml) for 24 h. The Matrigel was then dissolved on ice with 5 mM EDTA/phosphate buffer saline (PBS) for 90 min. The cells were then gathered, centrifuged (600 g/3 min), washed with PBS, fixed with pre-chilled 70% ethanol, and kept at −20°C for 20 min. Thereafter, ribonuclease (200 μg/ml, R6513: Sigma-Aldrich, St. Louis, MO, United States) was then used to treat the cells for 30 min at room temperature (RT). After that, the organoid cells were stained with propidium iodide (50 μg/ml, Sigma-Aldrich) in a dark environment for 30 min at RT. The organoid cells were then gently pipetted, dissociated, and strained over a 70 µm nylon mesh strainer. Data acquisition was carried out using flow cytometry (Guava easyCyst, Millipore) and the phase distributions were quantified by the connected software.

### 2.6 TUNEL staining

Analysis of apoptosis in the frozen sections of Chaga-treated DBCO was performed as mentioned earlier (Elbadawy et al., 2021b) using a DeadEnd Fluorometric TUNEL system (Promega) following the manufacturer’s guidelines. DBCO cells (1 × 10^5^/well) were cultured for 24 h. The organoid cells were then treated with Chaga (100 μg/ml) or DMSO and kept in a CO2 incubator for 72 h. Frozen sections were generated using Leica cryostat (Leica CM3050 S, Leica Biosystems) and preserved at −80°C until used for apoptosis assay. For TUNEL staining, the slides were washed with PBS for 5 min, fixed with 4% paraformaldehyde (PFA, Fujifilm Wako Pure Chemical Co.) for 15°min, permeabilized with 0.2% Triton X-100/PBS for 5°min, and fixed again in 4% PFA for 5 min. To assure the assay was successful, positive control slides (using DNase I, 0.5°U/well) were prepared in separate copulins and negative control ones (without rTdT enzyme) were also prepared. The organoid sections were then covered with 100 µl of equilibration buffer for 10 min at RT followed by 100 µl of rTdT incubation buffer and kept in a humidified chamber for 1 h at 37°C to allow the tailing reaction to begin. The reaction was then terminated by soaking the slides in SCC solution (2X) for 15 min at RT. After washing the slides three times with PBS for 5 min, sections were stained with propidium iodide/PBS (0.1%) for 15 min in a dark condition. The cell nuclei were counterstained with Hoechst/PBS (3 μg/ml in) for 5 min. The sections were then washed three times with deionized water for 5 min at RT, mounted with a drop of anti-fade solution, and sealed. The fluorescein12-dUTP-labeled DNA in the sections was visualized by a fluorescence microscope (BX61, Olympus) and several images were captured from different fields and quantified by ImageJ densitometry analysis software (National Institutes of Health, Bethesda, MD, United States).

### 2.7 Western blotting

Western blot analysis was performed to examine the protein expression levels of total and phosphorylated ERK. Briefly, organoids were seeded on a 24-well culture plate at 2 × 10^5^ cells/well. Twenty-4 h later, organoids were treated with Chaga (100 μg/ml), and DMSO was used for the control wells. Six, 12, 24, and 48 h later, treated organoids were harvested and mixed with RIPA lysis buffer (Sigma-Aldrich) including a 1% protease inhibitor cocktail (Sigma-Aldrich) to extract their protein. The protein content of lysates was quantified using a DC protein assay kit (Bio-Rad Laboratories, Hercules, CA, United States) and prepared for immunoblotting according to the standard procedures. Briefly, equal amounts of proteins (10 µg) were loaded on 10% SDS-PAGE gel (ATTO Co., Ltd., Tokyo, Japan) and electrophoresed. They were then transferred to a nitrocellulose membrane (Fujifilm Wako Pure Chemical Corporation). Blocking of membranes were carried out using 1X TBST with 5% w/v nonfat dry milk for 1 h at RT with gentile shaking. Thereafter, the membranes were treated with the diluted (in 1X TBST with 5% bovine serum albumin) primary antibodies [T-ERK (1:250) and P-ERK (1:250)] and incubated at 4°C overnight with gentle shaking. The membranes were then incubated with the secondary antibody for 1 h at RT. The EZ-Western Lumi Plus Kit (ATTO Co., Ltd.) was used to observe the emission. The band images were captured by a LAS-3000 image analyzer (Fuji Film, Tokyo, Japan) and quantified by an ImageJ densitometry analysis software (National Institutes of Health).

### 2.8 Quantitative real-time PCR

The effects of Chaga on cell cycle- and CSC-related signal pathways in DBCO were assayed using quantitative real-time PCR. Concisely, the organoids were cultured on a 24-well culture plate at 1 × 10^5^ cells/well. Twenty-4 h later, the organoid cells were treated with Chaga (100 μg/ml), or DMSO (for the control wells) for 24 h. Thereafter, organoids were then harvested, and the total RNA was generated using the NucleoSpin RNA kit (TAKARA, Tokyo, Japan) and converted to first-strand cDNA using the ReverTra Ace qPCR RT Kit (Toyobo Co., Ltd., Osaka, Japan) following the manufacturer’s protocols. Real-time PCR was carried out by the QuantiTect SYBR I kit (Qiagen, Hilden, Netherlands) and StepOnePlus Real-Time PCR system (Applied Biosystems, Waltham, Massachusetts, United States). The ^ΔΔCq^ method was used to quantify the data. Data analysis was carried out in triplicates and GAPDH was used as a control gene. The primers for *GAPDH*, *Cyclin A2, Cyclin D1, Cyclin E1, CDK4, CD44, c-MYC, SOX2*, and *YAP1* genes were used (Fasmac, Table 2).

**TABLE 2:** Primers for real-time quantitative PCR analysis of DBCO.

Gene	Primer	Sequence
*GAPDH*	Forward	5′-CGA​GAT​CCC​TCC​AAA​ATC​AA-3′
	Reverse	5′-TGT​GGT​CAT​GAG​TCC​TTC​CA-3′
*Cyclin-A2*	Forward	5′-TGA​GGG​CTA​TCC​TTG​TGG​AC-3′
	Reverse	5′-GGT​GCA​GCT​AGG​TCA​AAA​GC-3′
*Cyclin-D1*	Forward	5′-TGT​TTG​CAA​GCA​GGA​CTT​TG-3′
	Reverse	5′-TCA​TCC​TGG​CAA​TGT​GAG​AA-3′
*Cyclin E1*	Forward	5′-TCG​CAG​AGC​TTT​TGG​ATC​TT-3′
	Reverse	5′-GCA​CCA​TCC​ACT​TGA​CAC​AC-3′
*CDK4*	Forward	5′-CCC​CGT​CCA​GTA​CAG​ACA​GT-3′
	Reverse	5′-AGG​CAG​AGA​TTC​GCT​TGT​GT-3′
*CD44*	Forward	5′-CAA​CAC​AAA​TGG​CTG​GTA​CG-3′
	Reverse	5′-GTG​TGG​TTG​AAA​TGG​TGC​TG-3′
*C-MYC*	Forward	5′-GTT​ATC​TCG​CAA​ACC​CCA​GA-3′
	Reverse	5′-GGC​ATG​ATA​GCG​AAG​GGT​TA-3′
*SOX2*	Forward	5′-GCC​CTG​CAG​TAC​AAC​TCC​AT-3′
	Reverse	5′-GGA​GTG​GGA​GGA​GGA​GGT​AA-3′
*YAP1*	Forward	5′-CAC​AGC​ATG​TTC​GAG​CTC​AT-3′
	Reverse	5′-AGA​GGA​GGT​CTT​GGC​CAT​CT-3′

### 2.9 Mouse xenograft assay

The xenograft study was carried out according to the directions of the ‘Guide for the Care and Use of Laboratory Animals and approved by ethics committees of the Tokyo University of Agriculture and Technology (Approval number: R04-120). Six-week-old 8 male SCID mice (C.B-17/IcrHsd-Prkdcscid) were purchased from Sankyo Laboratory (Tokyo, Japan), housed under a specific pathogen-free environment, and allowed to acclimatize for 1 week before experimentation. To study the *in vivo* inhibiting effects of Chaga on tumor growth in mice, organoid cells (1 × 10^6^) were mixed with Matrigel/culture medium (50% v/v) and inoculated under the skin of the back of mice. After tumor growth (2 weeks after the organoid injection), mice were randomly divided into two groups (Chaga and vehicle groups), each of four mice. Chaga [6 mg/mouse/day (Arata et al., 2016)] or vehicle (1% β cyclodextrin) was orally administered by gavage for 4 weeks. Tumor dimensions were calibrated every week for 4 weeks of administration and their volumes (V) were evaluated as follows: V = 1/2 (L×W^2^), where L and W are the longest (length) and shortest (width) dimensions, respectively and the mean tumor volumes were calculated for Chaga and vehicle groups. Subsequently, mice were euthanized under isoflurane anesthesia and tumors were isolated, PBS-washed, weighed, fixed with PFA (4%), and embedded in paraffin. Four µm sections were prepared for hematoxylin and eosin (H&E) staining.

### 2.10 Histology (H&E staining)

Sections from Chaga- or vehicle-treated DBCO-derived xenograft were stained with H&E as mentioned previously (Elbadawy et al., 2019). Briefly, xenograft tissues were fixed at RT in 4% PFA/PBS for 2 h and embedded in paraffin. Sections preparation and staining steps were carried out following the standard procedures. Representative images were taken by light microscopy (BX-52).

### 2.11 Statistical analysis

Data are shown as means ± SEM. One-way analysis of variance (ANOVA) and Bonferroni’s test were used to conduct the statistical analyses and *p*-values of <0.05 were considered statistically significant.

## 3 Results

### 3.1 Effects of Chaga on cell viability of DBCO

**FIGURE 1:**
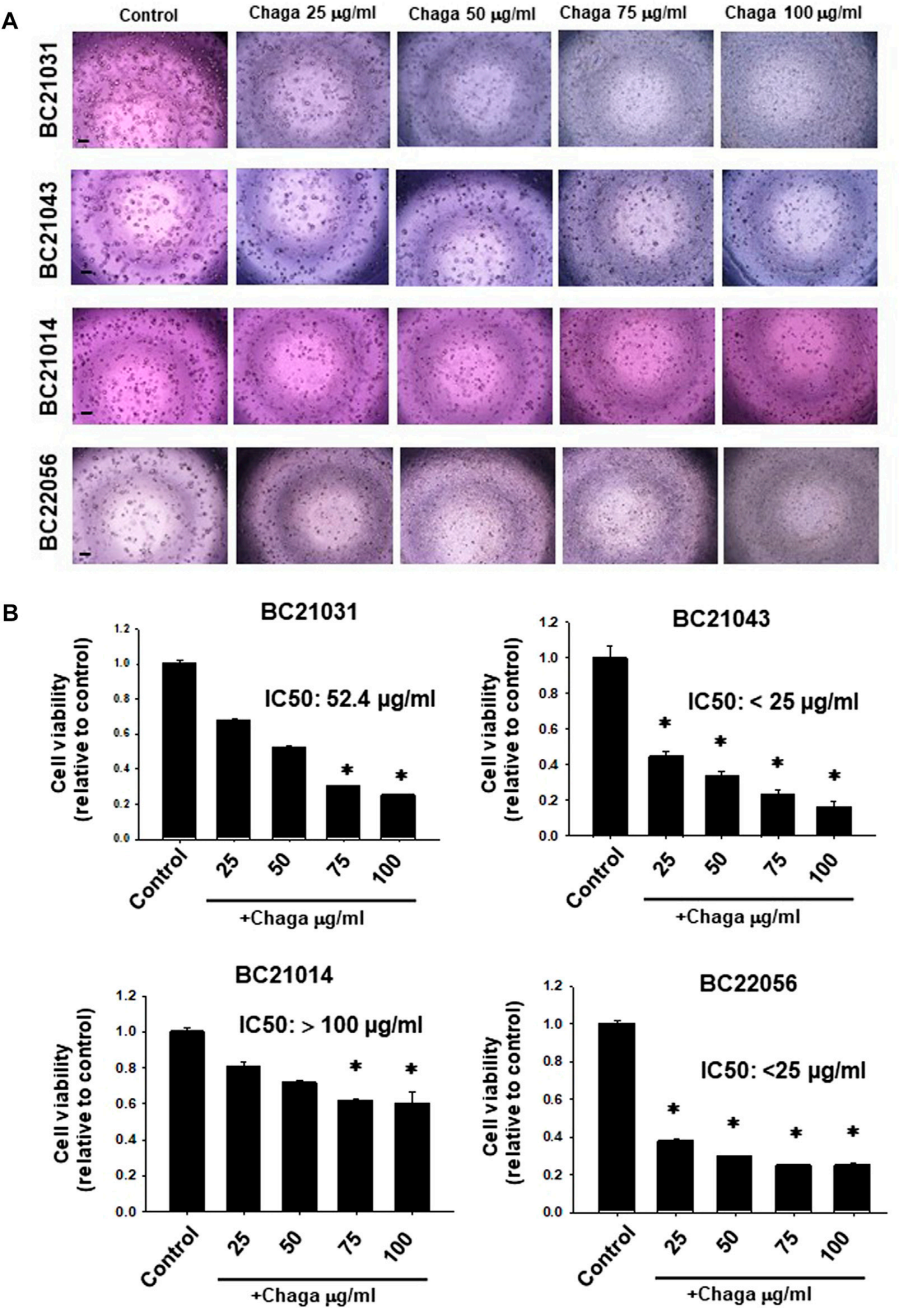
Effects of Chaga on cell viability of dog bladder cancer organoids (DBCO). Representative phase-contrast images (scale bar: 500 μm) of the sensitivity of four strains of DBCO (BC21031, BC21043, BC21014, and BC22056) to different concentrations (25, 50, 75, 100 μg/ml) of Chaga for 72 h using Prestoblue cell viability assay **(A)**, n = 3–6) and its quantification **(B)**. The value 1 on Y-axis represents the cell viability of each control. Results were expressed as mean ± SEM. *p < 0.05 vs. control.

In the previous study, we set up the method of DBCO and proposed that it become a new experimental model of human MIBC (Elbadawy et al., 2019). The anti-cancer effect of Chaga was reported previously on several cancer cell lines. We, therefore, examined the effects of Chaga on MIBC using DBCO. First, we checked the effects of Chaga on the cell viability of four different strains of DBCO. Chaga significantly diminished DBCO cell viability in a concentration-dependent (Figures 1A, B) and time-dependent way (Supplementary Figure S1). Interestingly, there was heterogeneity in the inhibition degree based on the organoid lineage (Figure 1B). Two strains were more sensitive than the other two strains (Figure 1B). These findings indicate that Chaga has an inhibitory effect on the DBCO.

### 3.2 Effects of Chaga on cell cycle and apoptosis of DBCO

**FIGURE 2:**
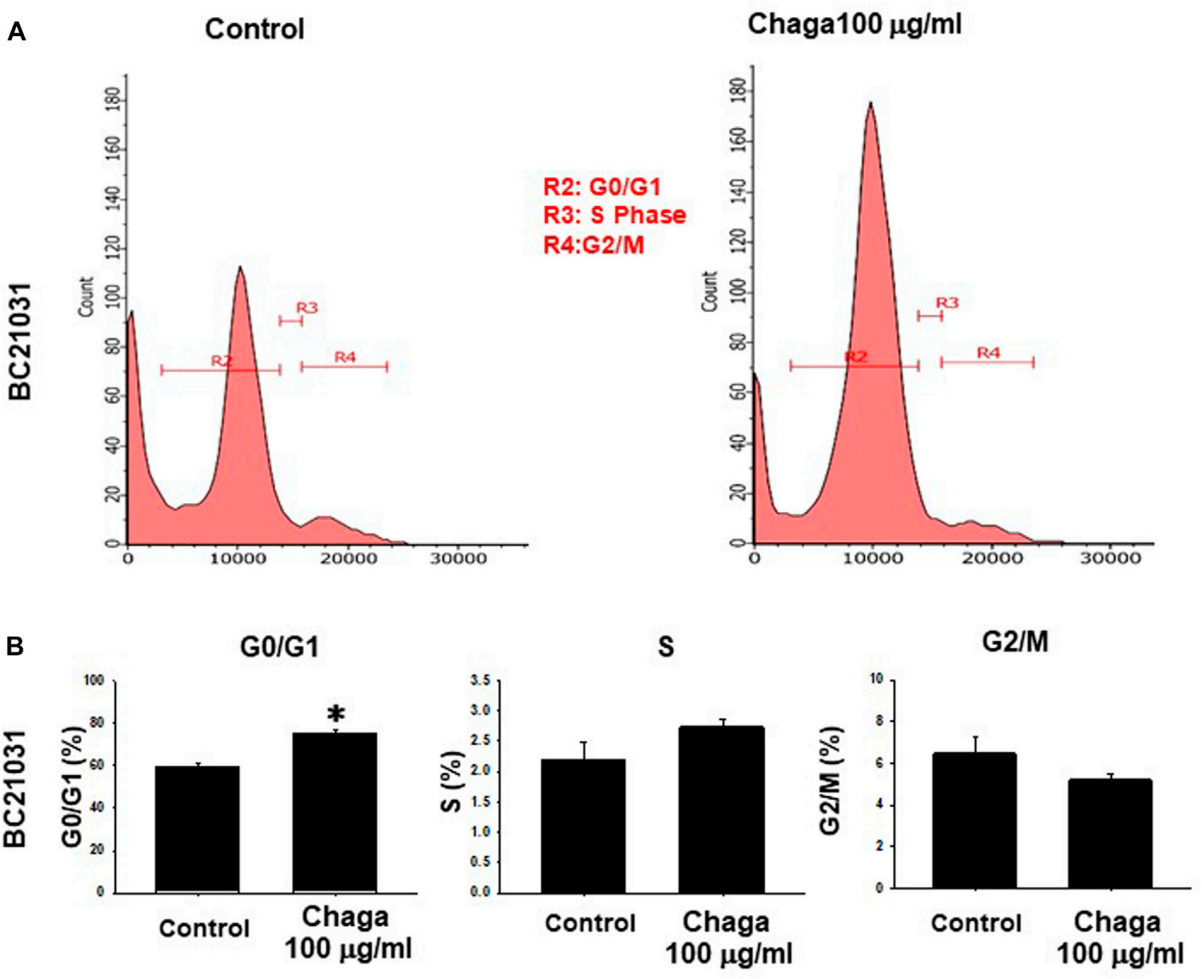
Effects of Chaga on cell cycle transition in DBCO. After DBCO cells were treated with Chaga (100 μg/ml for 24 h), they were stained with propidium iodide (PI). The distribution of cell cycle phases (G0/G1, S, and G2/M) of DBCO cells was analyzed by flow cytometry **(A)**. The population of G_0_/G_1_, S, and G_2_/M phases in DBCO was plotted and expressed as mean ± SEM **(B)**, n = 4). *p < 0.05 vs. control.

To explore the inhibitory mechanisms of Chaga on DBCO, we investigated whether Chaga influences the cell cycle of DBCO. Analysis of flow cytometry results demonstrated that the ratio of cells in the G0/G1 phase in DBCO was significantly elevated 24 h after Chaga treatment compared with the vehicle treatment. The proportion of cells in the S phase was also increased. On the other hand, the proportion of cells in the G2/M phase declined 24 h after Chaga treatment compared with the vehicle treatment (Figures 2A, B). These data indicate that Chaga treatment (100 μg/ml) for 24 h is arresting DBCO cells at G0/G1 phase. These arrested cells undergo killing upon prolonging the treatment duration for 72 h as evidenced by cell viability data (Figure 2).

**FIGURE 3:**
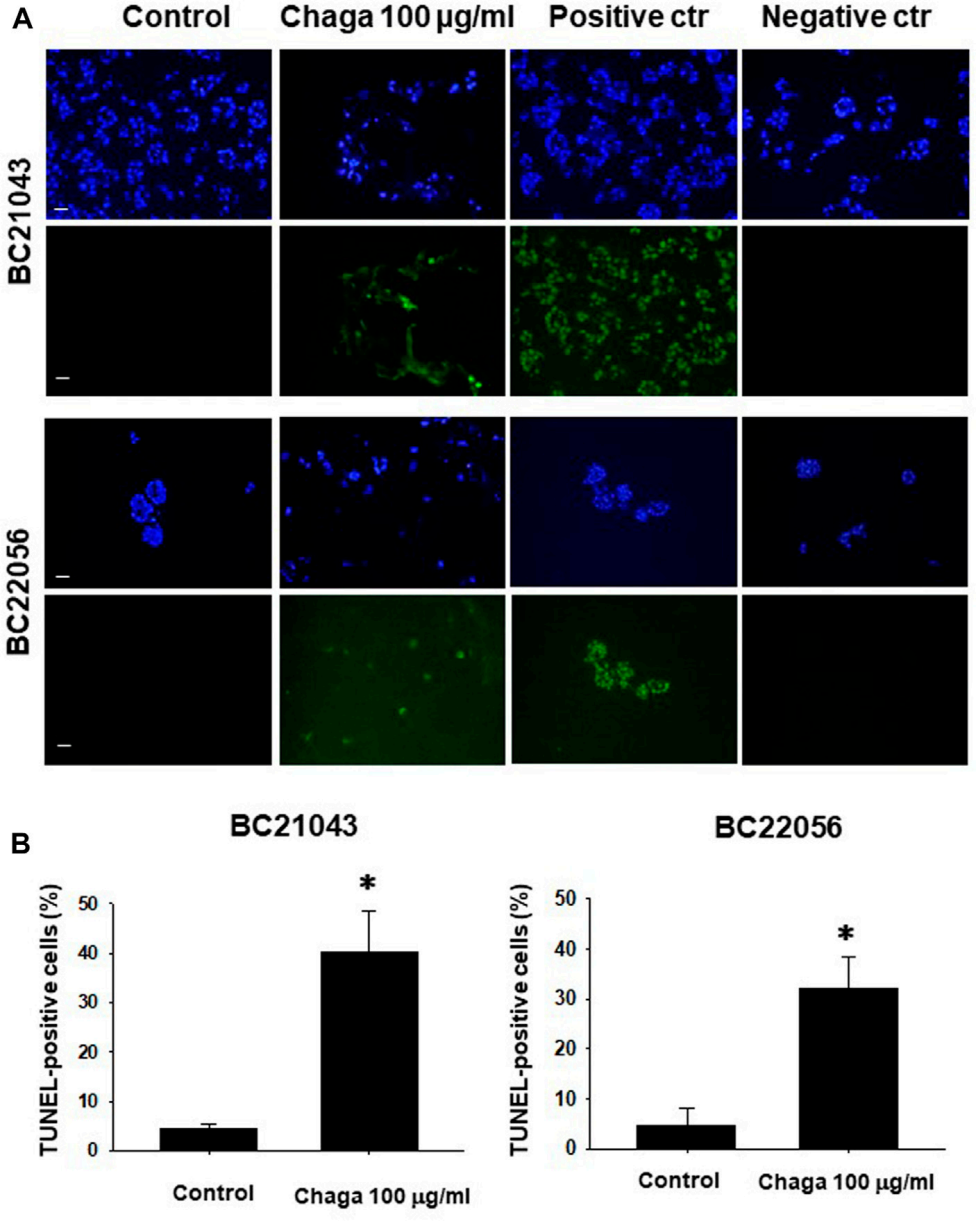
Effects of Chaga on apoptosis in DBCO. TUNEL staining of frozen sections (8 μm thickness) of two strains of DBCO (BC21043 and BC22056) treated with Chaga (100 μg/ml for 72 h). DNase-treated organoid cells were used as the positive control (n = 3). PBS-treated organoid cells were used as the negative control (n = 3). The green fluorescence area indicates apoptotic-positive organoid cells, and the blue DAPI staining shows intact DNA. Scale bar: 100 μm **(A)**. Quantification of apoptosis in Chaga-treated and non-treated cells (Control) was analyzed by ImageJ software **(B)**, n = 3). Results were expressed as mean ± S.E.M. *p < 0.05 vs. control.

Next, we examined whether treatment of DBCO with Chaga induces apoptosis. In TUNEL staining, the ratio of FITC-positive cells demonstrating apoptosis in the Chaga-treated DBCO was significantly higher compared with the vehicle treatment (Figures 3A, B). These data suggest that Chaga decreased the viability of DBCO by arresting cell cycle transition and inducing apoptosis.

### 3.3 Effects of Chaga on cell proliferation-related signals of DBCO

**FIGURE 4:**
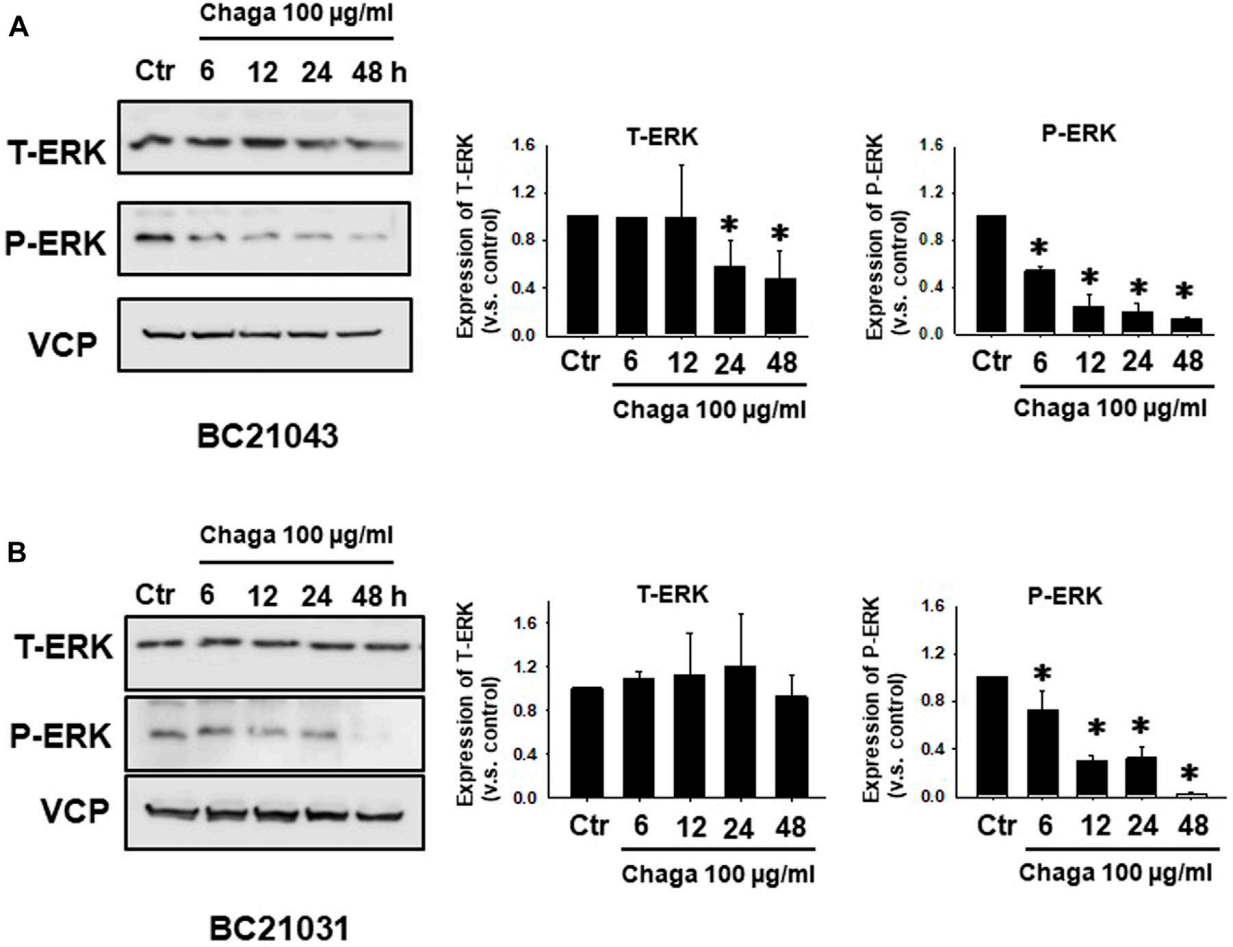
Effects of Chaga on activation of cell proliferation-related signaling in DBCO. Expression of phosphorylated ERK (P-ERK) and total ERK (T-ERK) in DBCO after Chaga treatment (100 μg/ml for 6, 12, 24, and 48 h) were determined by Western blot analysis **(A, B)**, (n = 3). The expression levels were quantified and plotted based on the ratio of expression level to control **(A, B)**, n = 3). Results were expressed as mean ± S.E.M. Equal protein loading was confirmed using total VCP antibody. * *p* < 0.05 vs. control.

**FIGURE 5:**
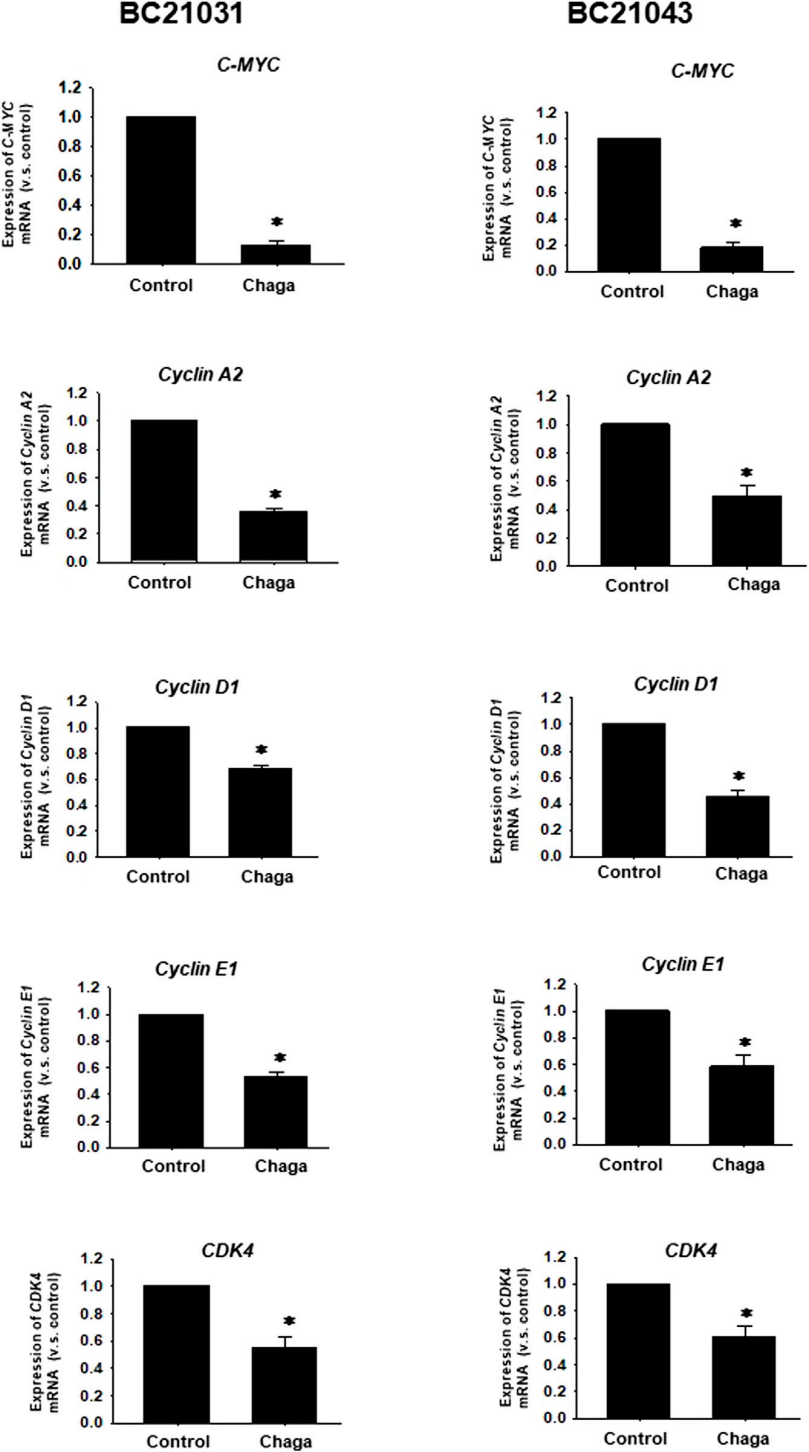
Effects of Chaga on mRNA expression levels of C-MYC and cell cycle-related genes in DBCO. mRNA expression of *C-MYC, Cyclin A2*, *Cyclin D1*, *Cyclin E1,* and *CDK4* was determined by quantitative real-time PCR. The expression level of each gene was quantified based on the ratio of expression level to *GAPDH* and shown as a fold increase relative to control (n = 4). Results were expressed as mean ± S.E.M. * *p* < .05 vs. control.

Next, the impact of Chaga on cell proliferation-related pathways of DBCO was assayed. Western blot analysis revealed that phosphorylation of ERK (P-ERK), a regulator of cell growth, was significantly declined at 6–48 h of Chaga treatment (100 μg/ml) compared with the vehicle treatment (Figures 4A, B, Supplementary Figure S2). Therefore, the impact of Chaga on the downstream signals of ERK cascade (C-MYC) and cell cycle pathways were further examined using real-time PCR and analysis of the data showed that expressions of *C-MYC, cyclin-A1, cyclin-D1, cyclin-E1,* and *CDK4* in DBCO were significantly decreased after 24 h of Chaga treatment compared with vehicle treatment (Figure 5). These findings propose that Chaga also targets the proliferation pathways of DBCO.

### 3.4 Effects of Chaga on stemness of DBCO

**FIGURE 6:**
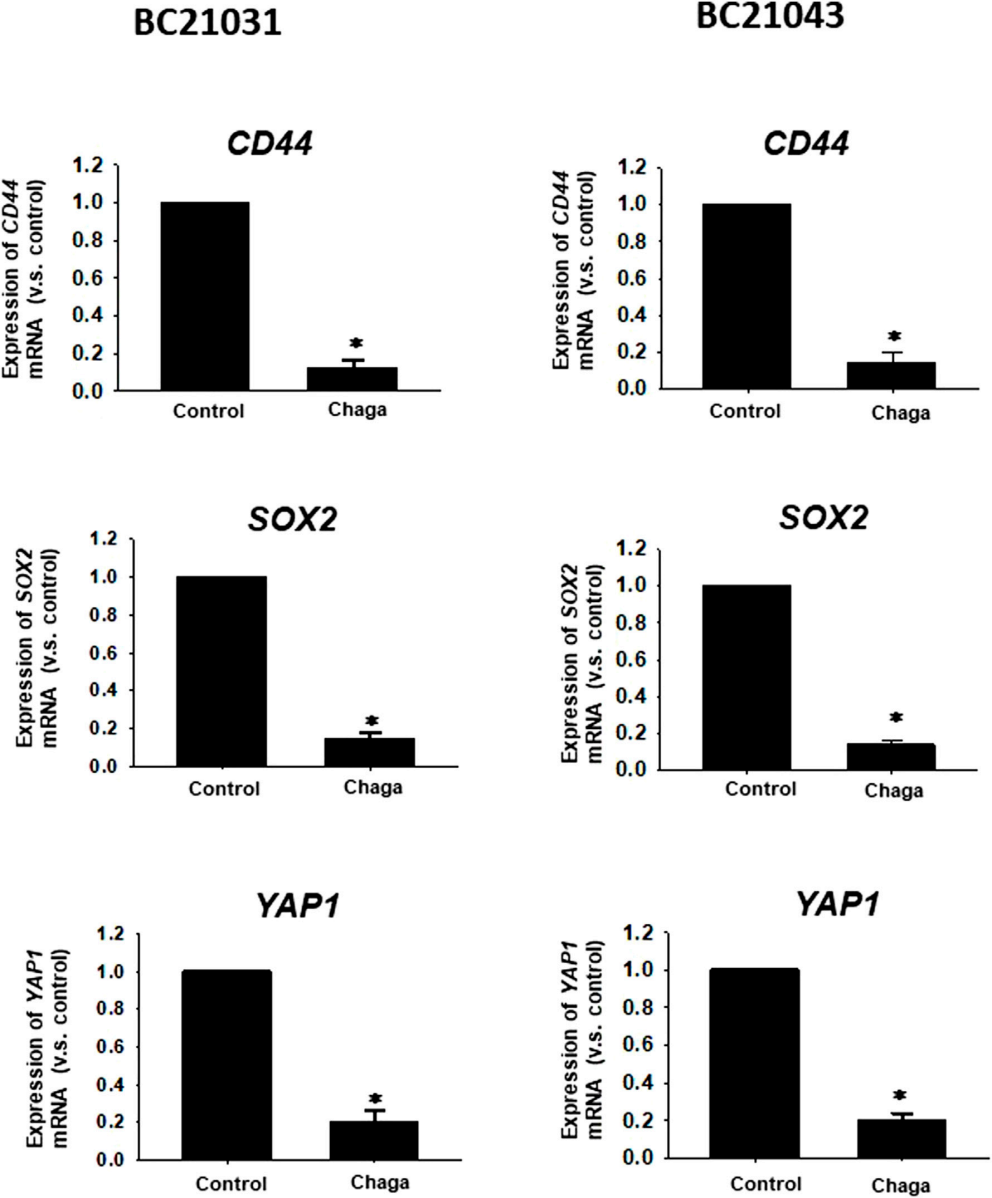
Effects of Chaga on stemness of DBCO. mRNA expression levels of *CD44*, *SOX2*, and *YAP1* in Chaga-treated (100 μg/ml for 24 h) DBCO were determined by quantitative real-time PCR. The expression level of each gene was quantified based on the ratio of expression level to *GAPDH* and shown as a fold increase relative to control (n = 4). Results were expressed as mean ± S.E.M. *p < 0.05 vs. control.

Since CSCs are implicated in the growth, migration, invasion, angiogenesis, anti-cancer drug resistance, and metastasis in MIBC, the influence of Chaga on the expression levels of three important CSC markers, *CD44*, *SOX2*, and *YAP1* which were upregulated in most of MIBC (Ciamporcero et al., 2016) were examined. Treatment of DBCO with Chaga (100 μg/ml/24 h) significantly decreased the level of *CD44*, *SOX2*, and *YAP1* mRNA expression (Figure 6). These results suggest that Chaga also targets the stemness pathways of DBCO.

### 3.5 Potentiating effects of Chaga on the sensitivity of DBCO to anti-cancer drugs

**FIGURE 7:**
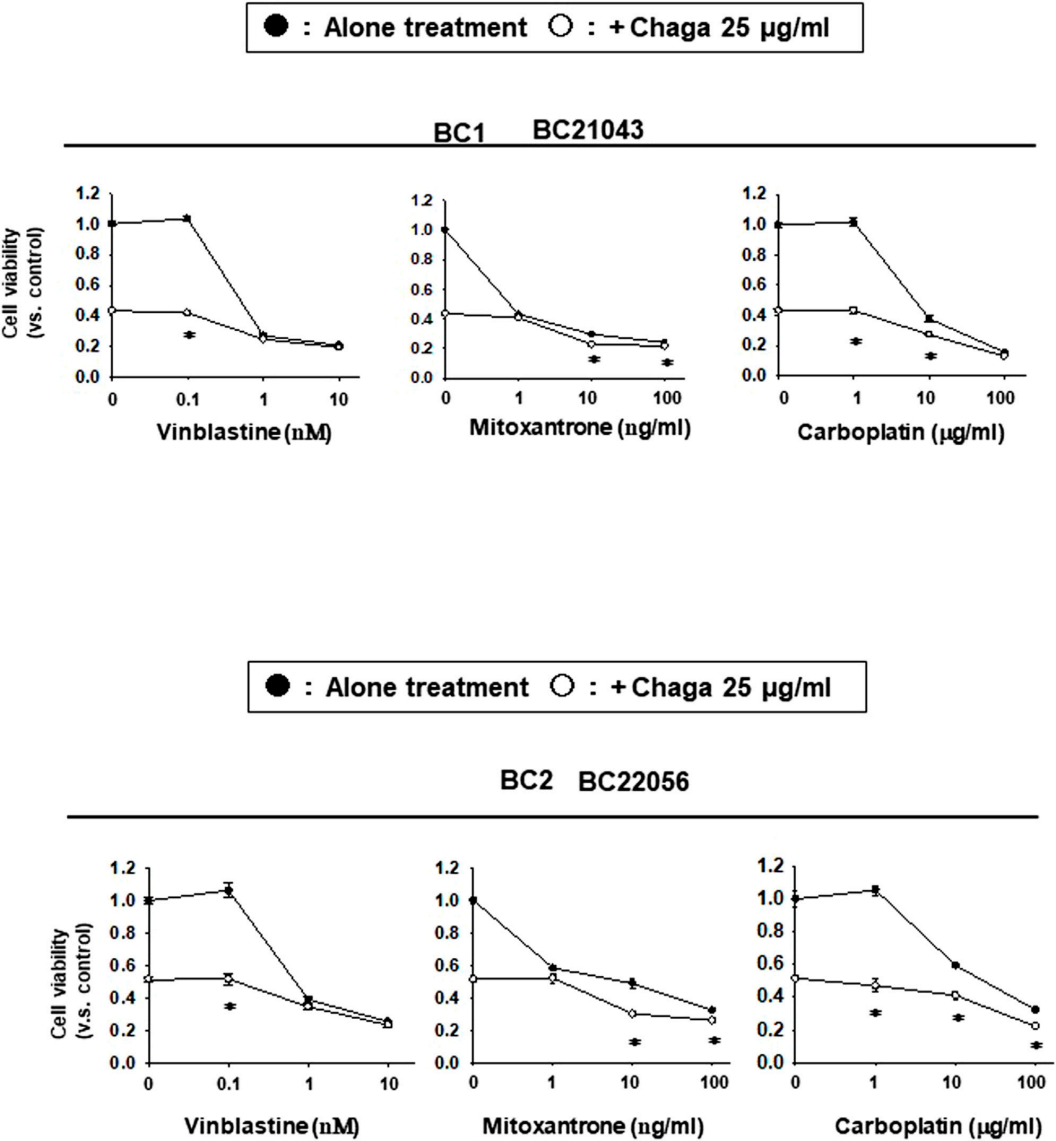
Potentiating effects of Chaga on the sensitivity of DBCO to anti-cancer drugs. Using two strains of DBCO (BC21043 and BC22056), the potentiating effects of Chaga on vinblastine (0.1–10 nM), mitoxantrone (1–100 ng/ml), or carboplatin (1–100 μg/ml) were verified. Cell viability was determined using the PrestoBlue kit, and the value 1 on Y-axis represents the cell viability of each control. The effect of Chaga alone (25 μg/ml) is shown on the Y-axis (opened circle between the values 0.4 and 0.6). The effect of cytostatic alone is shown in line with black circles and the effect of the combination is shown in line with white circles. Results were expressed as mean ± S.E.M (n = 3). * *p* < 0.05 vs. alone treatment.

Next, we investigated the synergistic potential of Chaga on the generally used BC anti-cancer drugs. The effects of common BC anti-cancer drugs were assayed alone or combined with a low concentration of Chaga (25 μg/ml) on the cell viability of DBCO. Chaga significantly potentiated the anti-cancer effects of vinblastine, mitoxantrone, and in DBCO, especially at their low concentrations (Figure 7). These data suggest the potential of Chaga not only to inhibit the cell viability of DBCO but also to potentiate the effects of anti-cancer drugs on BC.

### 3.6 Effects of Chaga on tumorigenesis of DBCO

**FIGURE 8:**
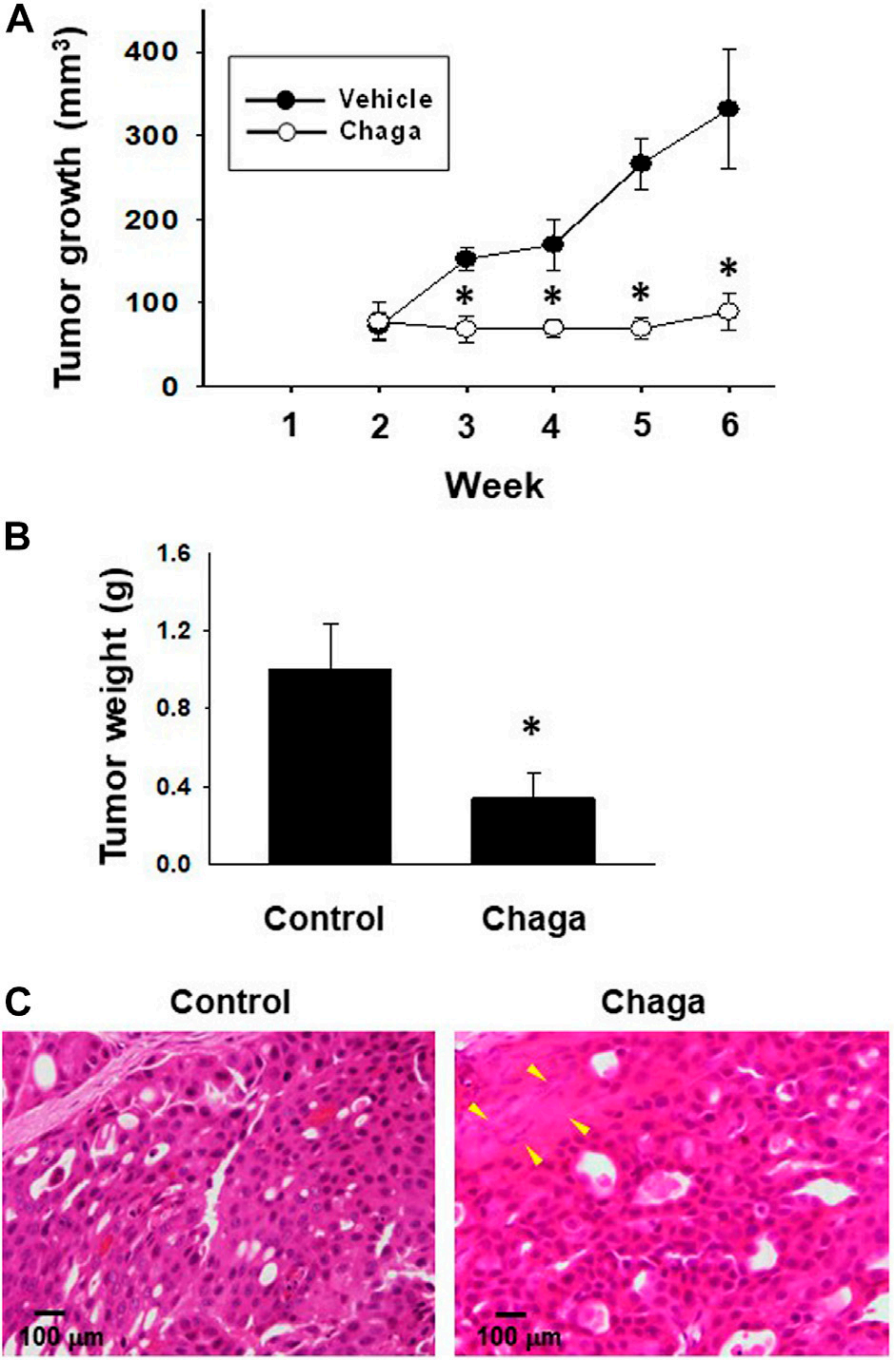
Effects of Chaga on tumor growth against xenografted DBCO in immunodeficient mice. After DBCO cells were subcutaneously inoculated into the back of SCID mice and tumors were formed, Chaga was administered to the mice at the concentration of 6 mg/kg for 4 weeks and the vehicle was administered at the same volume to the control group. Thereafter, the tumor volume was calibrated weekly for 4 weeks **(A)**, (n = 4). The tumor tissue of each group was dissected, weighed, and used for analysis. The tumor growth **(A)** and weight **(B)** between Chaga- or vehicle-administered mice were analyzed and graphed. Results were expressed as mean ± S.E.M. *p < 0.05 vs. control. Representative H&E staining images of the tumor tissue sections from Chaga- or vehicle-administered mice **(C)**. Scale bar: 100 μm.

To evaluate the *in vivo* anti-cancer effects of Chaga, we carried out a xenograft experiment of DBCO. After tumor growth, Chaga was given to mice for 4 weeks. The tumor volumes in the Chaga-treated group were significantly decreased on the 7th, 14th, 21st, and 28th day of Chaga administration compared to the vehicle-administered group (Figure 8A). Further, tumor weights in the Chaga-treated group were also significantly lower than those of vehicle-administered group (Figure 8B). In histopathological examination, necrosis was observed in tumor sections of Chaga-treated group (Figure 8C).

## 4 Discussion

**FIGURE 9:**
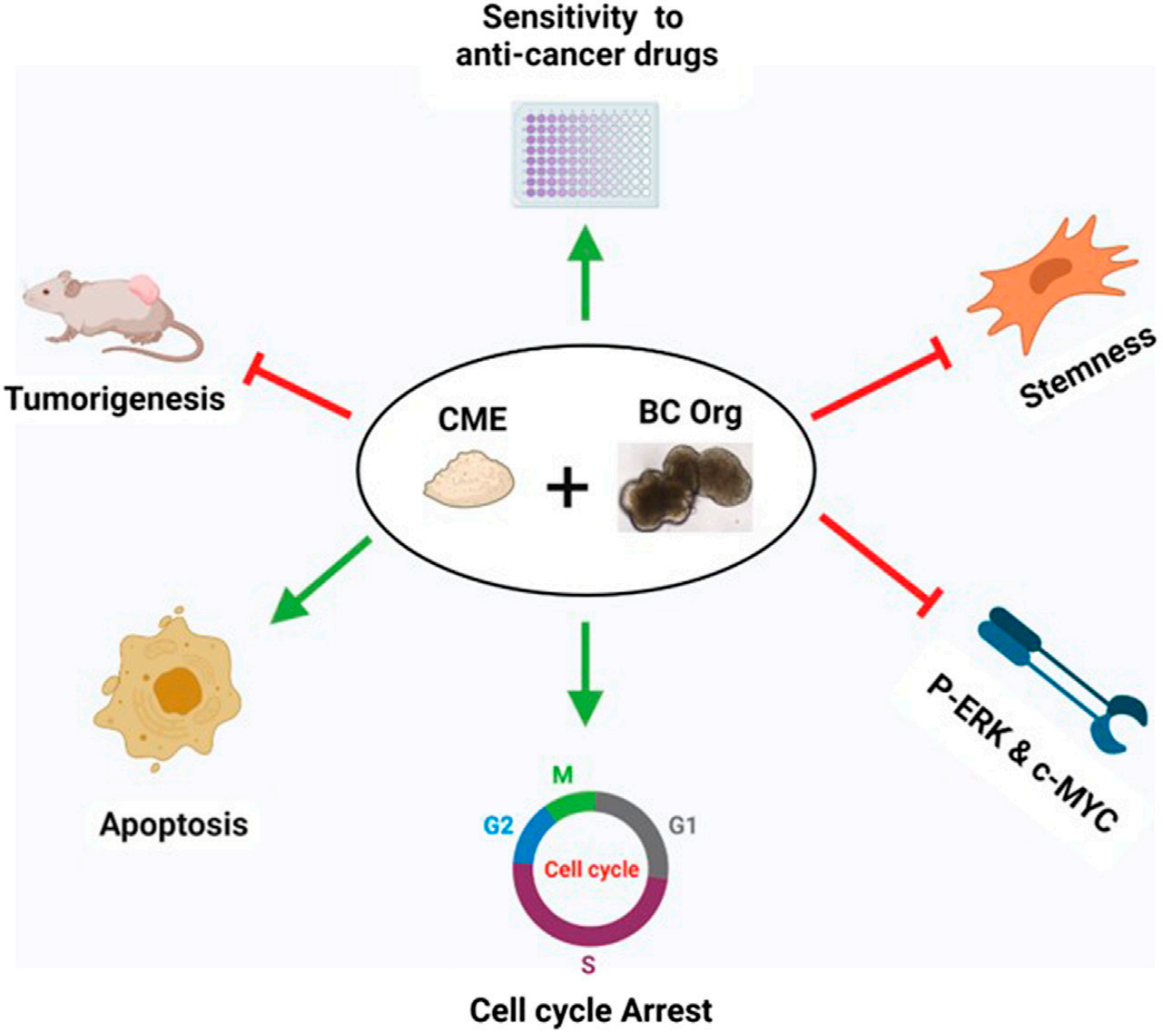
Summary of the effects of Chaga on DBCO. Chaga suppressed the survival of organoids by arresting the cell cycle, inducing apoptosis, targeting stemness, and decreasing the phosphorylation of ERK and expression of its downstream signal molecule, C-MYC, and Cyclins. Chaga also enhanced the action of the commonly prescribed anti-cancer drugs for BC. Further, the effect of Chaga has been verified *in vivo*.

In the current investigation, we examined the effects of Chaga on DBCO. The key results are as follows: 1) Chaga decreased the cell viability of DBCO in a concentration-based way (Figure 1), 2) Chaga upregulated the G0/G1 phase and downregulated the G2/M phases of the cell cycle in DBCO (Figures 2A, B), 3) Chaga triggered apoptotic alterations in the DBCO (Figures 3A, B), 4) Chaga diminished the phosphorylation of ERK as well as expression of its downstream signaling molecules, *C-MYC* and *Cyclins* in DBCO (Figures 4, 5), 5) Chaga reduced expression of CSC markers, *CD44*, *SOX2*, and *YAP1* in DBCO (Figure 6), 6) Chaga potentiated the effect of BC common anti-cancer drugs, vinblastine, mitoxantrone, and carboplatin in DBCO (Figure 7), 7) Chaga diminished the development of DBCO-derived xenografted tumors in mice and induced necrotic spots in the xenografted tumors (Figure 8). Collectively (Figure 9), these data proposed the value of Chaga as a promising natural supplement with anti-cancer drugs for MIBC.

In the present study, we evaluated the anti-tumor effects of Chaga on DBCO *in vitro* and *in vivo*. Previously, the *in vitro* cytotoxic outcomes of Chaga have been explored in lung adenocarcinoma (Baek et al., 2018), sarcoma (Chung et al., 2010), colon cancer (Lee et al., 2009), melanoma (Youn et al., 2009) as well as hepatocellular carcinoma (Youn et al., 2008). Despite growing evidence of anti-tumor potentials exhibited by the Chaga and its components (Shashkina et al., 2006; Song et al., 2013), the underlying mechanisms are still not well understood. This may be due to most of these studies being conducted on cell line models (Szychowski et al., 2021). Our study is the first to investigate the anti-cancer effects of Chaga on BC using DBCO, which is a novel experimental model for MIBC in humans (Elbadawy et al., 2019). In the present study, the anti-cancer effects of Chaga in DBCO were mediated via targeting numerous signaling pathways such as cell cycle, CSC, and apoptosis pathways which were similarly reported in various cancer cell lines (Youn et al., 2008; Youn et al., 2009; Baek et al., 2018; Lee et al., 2021).

In the current study, Chaga significantly inhibited the cell viability of DBCO in a concentration-dependent way with a difference in the sensitivity among the four strains used. Similarly, the antiproliferative effect of Chaga on morphology and cell proliferation of hepatoma cells (Hep3B and HepG2) was evaluated and showed a dose-dependent cytotoxic effect with a difference in sensitivity to Chaga between the two strains (Youn et al., 2008). The same findings were reported in human lung carcinoma, colon adenocarcinoma, and rat glioma cell lines (Lemieszek et al., 2011; Kang et al., 2015). Chaga also decreased cell migration and altered their morphology (Lemieszek et al., 2011). These data on cell lines corresponded with our findings in DBCO verifying the anti-tumor potential of Chaga.

In the current study, Chaga significantly increased the G0/G1 and decreased G2/M cell cycle phases (Figure 2), downregulated cyclins expressions (Figure 5), and induced apoptosis (Figure 3) in DBCO. Cyclin D1 works to bind CDK4 with CDK6 to produce an active complex that activated Rb protein at Ser780 and regulates the switch from G1 to S phase (Hernando et al., 2004). Previous data revealed that Chaga could induce cell cycle arrest and apoptotic changes in several cancer cell lines. In hepatoma cells (HepG2 and Hep3B) and melanoma B16-F10 cells, Chaga inhibited their growth by motivating cell cycle arrest at the G0/G1 phase and apoptosis (Youn et al., 2008; Youn et al., 2009). Also, in human lung adenocarcinoma cell lines with different p53 statuses (A549, H1264, H1299, and Calu-6), Chaga reduced their cell viability by causing apoptosis followed by caspase-3 cleavage (Baek et al., 2018). In human colorectal cancer cell lines (HCT116, HT-29, SW620, and DLD-1) ergosterol peroxide derived from Chaga, induced cell cycle arrest by reducing transcription of c-Myc, CDK-8, and cyclin D1 with increasing apoptosis induction (Kang et al., 2015). *In vivo*, it also induced a dramatic decline in the Ki67-positive cells and a raise in the TUNEL staining of colonic tumor epithelial cells in mice (Kang et al., 2015). In addition, Chaga exhibited cytotoxic effects through apoptosis induction in breast adenocarcinoma and murine lung tumors (Arata et al., 2016; Zhang et al., 2018). These data collectively indicate that Chaga targets cell cycle and apoptosis pathways to inhibit the growth and proliferation of DBCO.

It has been noted that ERK, a signal involved in the Ras/Raf/MEK/ERK pathway that regulates cell differentiation and proliferation, is overactivated in several malignancies, including BC (Elbadawy et al., 2021b). In the current investigation, Chaga dramatically reduced the activation of ERK and the expression of its downstream signals, C-MYC and cyclins (Figures 4, 5). It was revealed that C-MYC is a key player in bladder CSC-related signaling and treatment resistance, and is identified as a key therapeutic target for several malignancies (Elbadawy et al., 2021b). Chaga-derived ergosterol peroxide was shown to decrease the transcription of C- Myc, cyclin D1, and CDK-8 and ultimately reduced the growth of colonic tumors in mice (Kang et al., 2015). These data imply that Chaga decreased the cell viability of DBCO through the downregulation of ERK/c-MYC/cyclins signals.

CSCs are a few cell populations that can launch and drive cancer progression and conserve cellular heterogeneity, self-renewal, and differentiation (Abugomaa et al., 2020). CSCs are fundamentally accountable for tumor metastasis, recurrence, and resistance to chemotherapy. Hence, targeting these cells with novel natural supplements with low side effects on normal cells like Chaga (Lemieszek et al., 2011) is necessary for the eradication of cancer. In BC, numerous CSC surface markers have been detected as responsible for BC development, metastasis, resistance to therapy, and recurrence (Li et al., 2017). Among them are CD44, SOX2, YAP1, and C-MYC signaling pathways (Li et al., 2017). Therefore, to combat the issues of BC’s resistance to therapy, metastasis, and recurrence, great focus has been given to the use of phytochemicals with unique CSC-targeting characteristics in combination with conventional anti-cancer drugs (Fulda, 2010; Orsolic et al., 2016; Su et al., 2016; Talib et al., 2020). In the present study, treatment of DBCO with Chaga significantly reduced the mRNA expression of CSC markers, *CD44*, *SOX2*, *C-MYC*, and *YAP1* (Figures 5, 6). Similarly, the combinational treatment of breast cancer cell lines, SK-BR-3, MCF-7, and MDA-MB-231 cells with various levels of tamoxifen, lapatinib, and doxorubicin, respectively, and Chaga exhibited synergistic or additive cytotoxic effects (Lee et al., 2021). These synergistic or additive cytotoxic effects occur because the ways that Chaga and anti-cancer drug produces their antitumor effect are different. This might lower the probability of developing resistance to traditional anti-cancer drugs in the therapy of BC.

In the present study, Chaga oral administration significantly reduced volumes and weights of DBCO xenografted tumors in mice (Figure 8). Similarly, Arata et al. (2016) demonstrated that a constant intake of the *Inonotus obliquus* aqueous extract induced a 60% tumor reduction and a 25% decline in the number of nodules in metastatic tumors compared with the control group in mouse models of Lewis lung carcinoma. In another *in vivo* study, ergosterol peroxide derived from Chaga suppressed the growth of tumors in the colon of Azoxymethane/Dextran sulfate sodium-treated mice with induction of apoptosis (Kang et al., 2015). Also, Lee et al. (2021) reported that Chaga oral administration effectively inhibited tumor growth in 4T1 tumor-bearing BALB/c mice. These data corresponded with our confirmed effects of Chaga to decrease tumor growth *in vivo*.

The anti-tumor potential of Chaga in the present study and previous investigations might be attributed to its phytochemical constituents of triterpenoids, small phenolic compounds, polysaccharides, and ergosterol peroxide as bioactive components (Kang et al., 2015; Zhao et al., 2015; Zhao et al., 2016). However, the biological activity of Chaga against BC, and the underlying molecular mechanism should be further checked and fully interpreted using other models to prop its therapeutic use in the therapy of BC.

In conclusion, we for the first time examined the antitumor potentials of Chaga on DBCO. Chaga inhibits DBCO by arresting the cell cycle, triggering apoptosis, decreasing stemness condition, and thus affecting cell proliferation (Figure 9). It was also noted that Chaga potentiated the effects of the common anti-cancer drugs used for BC therapy. Hence, the coadministration of Chaga with these anti-cancer drugs might decrease their dosages and thus lower their side effects and toxicities in BC-diseased patients. Further, the *in vivo* anti-tumor effect of Chaga has been confirmed against DBCO-derived xenograft in mice. These findings provide some molecular explanation for the anti-tumor potential of Chaga and further support its application for therapeutic intervention in BC. However, the biological activity of Chaga against BC, and the underlying molecular basis should be further addressed and fully elucidated using other models of BC to support its therapeutic application for BC. Also, further evaluation of the dosage is necessary. Thus, in the future, Chaga is expected to be co-administered as a natural supporting supplement with adjuvant chemotherapies to minimize chemoresistance and limit recurrence and metastasis in BC.

## Data Availability

The original contributions presented in the study are included in the article/Supplementary Material, further inquiries can be directed to the corresponding authors.
