# Supplementary figures and images for: Anti-cancer activity of Chaga mushroom (Inonotus obliquus) against dog bladder cancer organoids
Source: Front Pharmacol. 2023 Apr 19;14:1159516. doi: 10.3389/fphar.2023.1159516 (PMC10154587; doi:10.3389/fphar.2023.1159516)

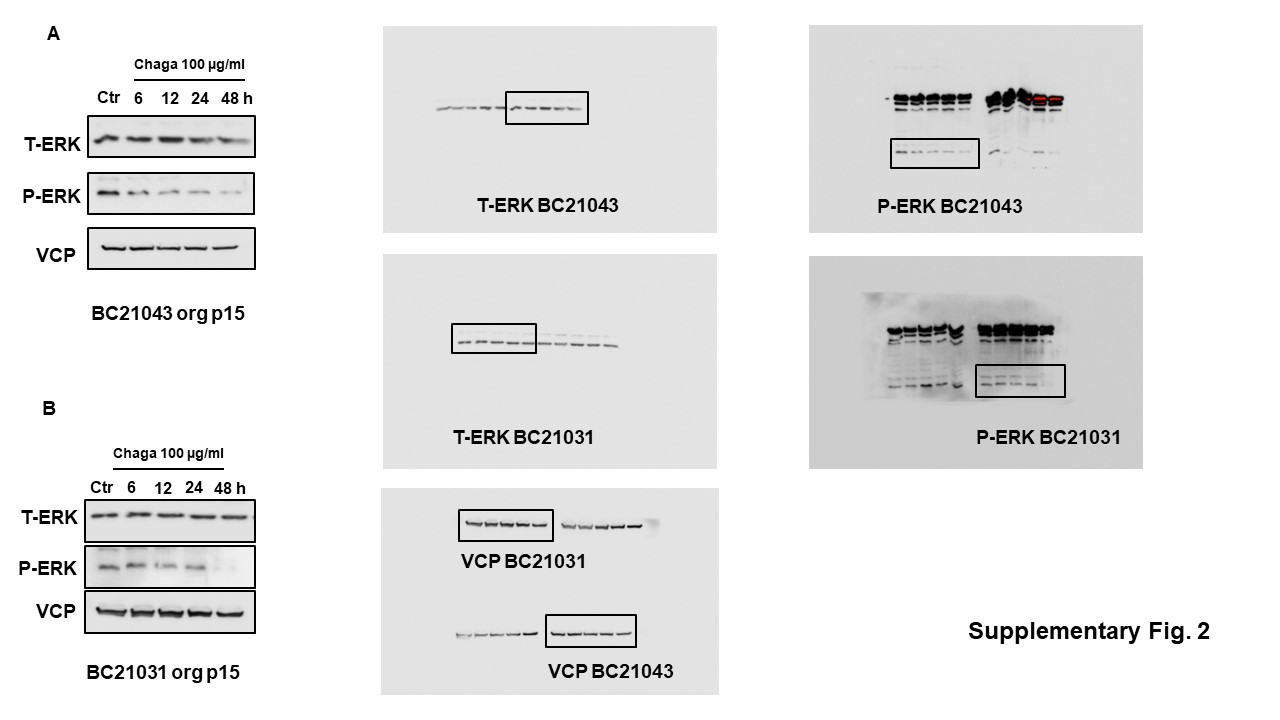

Supplement: Supplementary file 3 [file Image2.jpeg]

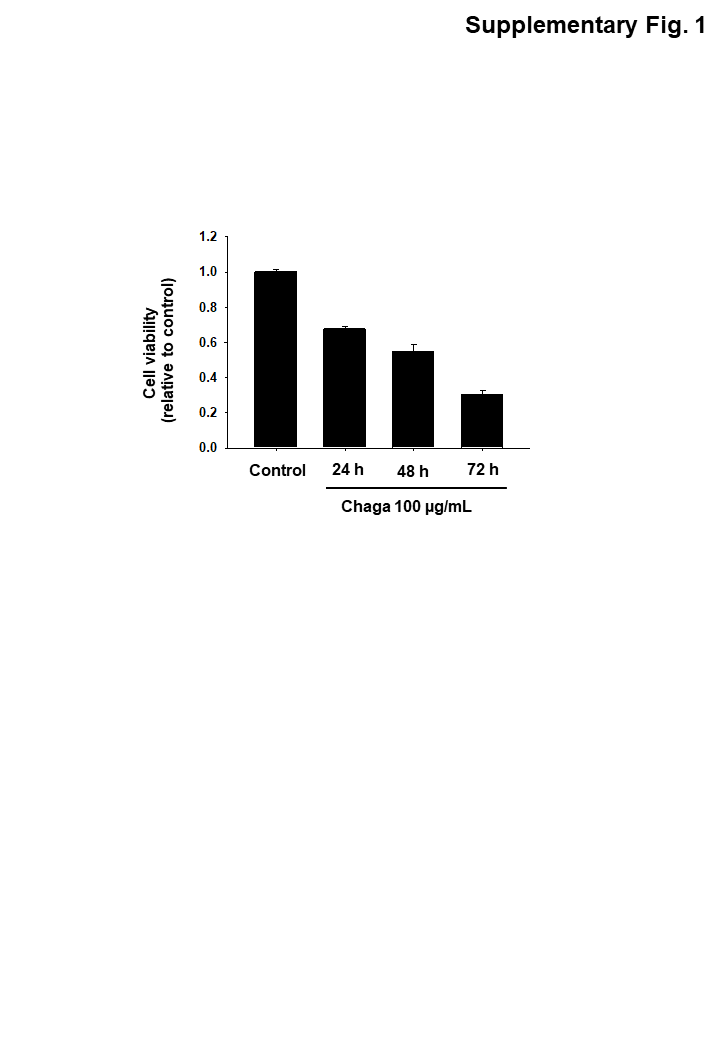

Supplement: Supplementary file 4 [file Image1.tif]
