# Supplementary material for: Anti-cancer activity of Chaga mushroom (Inonotus obliquus) against dog bladder cancer organoids
Source: Front Pharmacol. 2023 Apr 19;14:1159516. doi: 10.3389/fphar.2023.1159516 (PMC10154587; doi:10.3389/fphar.2023.1159516)
Supplement: Supplementary file 6 [file DataSheet1.docx]

**Supplementary figure legends**

**Supplementary Fig. 1.** Time-dependent effects of Chaga on cell viability of dog bladder cancer organoids (DBCO). After DBCO cells were treated with Chaga (100 μg/ml for 24, 48, 72 h), the cell viability was evaluated by Prestoblue cell viability assay (n=3). The value 1 on Y-axis represents the cell viability of each control. Results were expressed as mean ± SEM. **P<*0.05 vs. control.

**Supplementary fig. 2.** Row blot of western blotting data in Fig. 4.
